# Rare Case of Multiple Perirenal, Extra-Adrenal Myelolipoma: Case Report, Current Management Options, and Literature Review

**DOI:** 10.1155/2021/6614641

**Published:** 2021-04-13

**Authors:** Goran Benko, Andrina Kopjar, Marin Plantak, Danijel Cvetko, Vicko Glunčić, Anita Lukić

**Affiliations:** ^1^Department of Urology, Varaždin General Hospital, I. Meštrovića 1, Varaždin, Croatia; ^2^Department of Pathology, Varaždin General Hospital, I. Meštrovića 1, Varaždin, Croatia; ^3^Department of Radiology, Dubrava Clinical Hospital, Av. Gojka Šuška 6, Zagreb, Croatia; ^4^Department of Anesthesia, Advocate Illinois Masonic Medical Center, 836 W Wellington Ave, Chicago, IL 60657, USA; ^5^Department of Anaesthesiology, Reanimatology, and Intensive Care, Varaždin General Hospital, I. Meštrovića 1, Varaždin, Croatia

## Abstract

Extra-adrenal myelolipomas are rare, asymptomatic entities, although large tumors may cause local symptoms or hemorrhage. When these lesions occur outside the adrenals in the retroperitoneum, they are radiographically easily confused with both primary and secondary retroperitoneal tumors, which tend to be aggressive. Although myelolipomas are benign and can be managed conservatively, if malignancy is suspected, a surgical procedure is an option. We report a case of a 68-year-old patient with multiple perirenal extra-adrenal myelolipomas. Initial abdominal ultrasound reviled an inhomogeneous mass surrounding the left kidney. Subsequent CT examination of the abdomen showed four separate, extrarenal, well-circumscribed, round-shaped, fat-containing retroperitoneal tumors. Given the significant size of the masses, that compressed major abdominal vessels and the suspicion of liposarcoma, a surgical excision of the lesions was performed. The tumors were easily separated, all surrounding structures were spared, and they were removed completely. Histologically, all masses consisted of hematopoietic and mature fat tissue and the final diagnosis was extra-adrenal myelolipoma. The patient was released from the hospital 7th day after surgery in good condition and at his baseline. Since myelolipomas are, by definition, nonfunctional benign tumors, there was no need for further follow-up. The radiological evaluation and fine needle biopsy are usually sufficient to establish the diagnosis, but in some cases of well-differentiated liposarcoma, the differentiation between myelolipoma and liposarcoma can be challenging. Therefore, considering that myelolipomas and liposarcomas have opposite prognoses, which affects the surgeon's decision on the extent of surgical procedure and further treatment, we also emphasize the importance of intraoperative assessment of the tumor, both by the surgeon and by intraoperative pathology consultation.

## 1. Introduction

Myelolipomas are rare tumorous mixtures of adipose and relatively normal trilineage hematopoietic tissue. They usually occur as adrenal tumors (incidence is estimated at lower than 0.1%.), but extra-adrenal myelolipomas have also been described, occurring as head and neck, chest, intra-abdominal, or pelvic masses in up to 15% of all cases (Table 1) [1–4]. Perirenal extra-adrenal myelolipomas are especially rare and occur as single retroperitoneal perirenal masses [5].

Both adrenal and extra-adrenal myelolipomas are typically asymptomatic, although large tumors may cause local symptoms or hemorrhage [6]. Tumors could vary in size and often exceed 10 cm in diameter.

Although adrenal myelolipomas can be managed conservatively, when myelolipomas occur as large multiple retroperitoneal masses, they are radiographically easily confused with both, primary or secondary retroperitoneal tumors. Those could be aggressive neoplasms, such as liposarcoma, requiring different treatment [6].

In this report, we pinpoint the possibility that, although extremely rare, myelolipomas can occur as large, multiple, retroperitoneal, perirenal masses that could be initially mistaken for aggressive neoplasms.

## 2. Case Report

A 68-year-old man with lower urinary tract symptoms (LUTS) and well-controlled hypertension was referred to the department of urology. Initial abdominal ultrasound detected an inhomogeneous mass surrounding the left kidney. Complete laboratory exams were normal.

**Figure 1:**
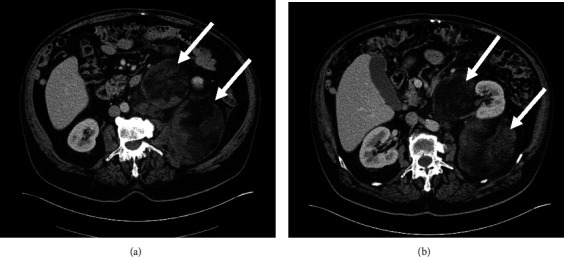
CT scan with multiple retroperitoneal myelolipomas (arrows).

Subsequent CT examination of the abdomen reviled four separate, extrarenal, well-circumscribed, round-shaped, and fat-containing retroperitoneal masses (12.0 × 12.0 cm, 12.0 × 8.0 cm, 6.0 × 3.5 cm, and 5.0 × 4.5 cm). Due to the volume of the masses, the aorta and inferior vena cava were compressed, as well as branches of the superior mesenteric artery. There was no hydronephrosis, and suprarenal glands and other organs appeared normal (Figures 1(a) and 1(b)). Based on the radiological findings, the initial differential diagnosis was liposarcoma.

**Figure 2:**
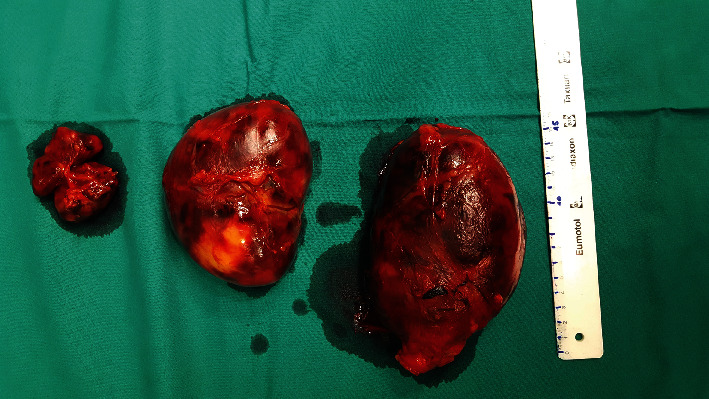
Gross appearance of surgically removed myelolipomas.

Given the significant size of the masses that compressed the major abdominal vessels and the suspicion of liposarcoma, a decision was made to surgically excise the lesions. The surgical procedure was performed using an open transabdominal approach, under general anesthesia. Tumors were soft and well encased by a thin capsule, intimately attached to the renal adipose capsule, ureter, and abdominal aorta. They were easily separated, all surrounding structures were spared, and the tumors were completely removed (Figure 2). Both surgical procedures and anesthesia were uneventful, with no significant blood loss.

**Figure 3:**
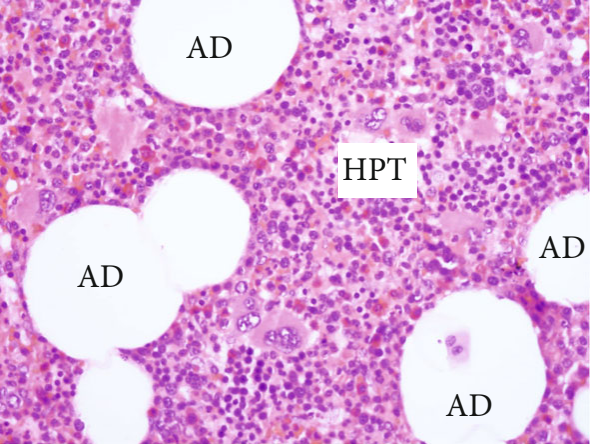
Microscopic appearance of myelolipoma with normal adipocytes (AD) and normal trilineage hematopoietic tissue (HPT) (hematoxylin-eosin staining, original magnification 40x).

Gross pathology revealed encapsulated, well-defined, soft masses measuring 11.5 × 8.5 × 6.0 cm, 15.0 × 10.0 × 6.5 cm, 6.0 × 5.5 × 3.5 cm, and 2.0 × 2.0 × 2.0 cm. Histologically, all the masses consisted of hematopoietic and mature fat tissue (Figure 3). The dominant tissue component was granulopoietic cells in various developmental stages, with the normal morphology of the megakaryocyte lines. There was no suprarenal gland tissue in the tumor, so the definitive diagnosis was extra-adrenal myelolipoma.

The patient was released from the hospital 7th day after the surgery, in good condition and at his baseline. Since myelolipomas are, by definition, nonfunctional benign tumors, there was no need for any further follow-up.

## 3. Discussion

The vast majority of myelolipoma cases have been presented as a single mass. They are asymptomatic, incidentally found on imaging studies, and can be managed conservatively [7, 8]. On ultrasonography, they present as a hyperechoic mass if the tumor is predominantly fatty, or as a hypoechoic mass, if the tumor is composed of myeloid cells. Small areas of hemorrhage within the mass can eventually calcify [9]. Most tumors are unilateral and vary in size from 2 to 26 cm at the time of diagnosis, but bilateral and even multifocal tumors have been described [5]. Spontaneous rupture of myelolipomas has been reported, usually when the tumor exceeded 10 cm.

Usually, extra-adrenal myelolipoma can be diagnosed by CT or MRI scans with great certainty [5, 7], and additional confirmation can be obtained by fine needle biopsy under ultrasound and/or CT guidance. Even so, the differentiation between (extra)adrenal myelolipoma and other fat-containing retroperitoneal tumors could be difficult. Indeed, the majority of fat-containing tumors are well-differentiated liposarcomas [10], which can be misleading and can represent a challenge for radiologists, since many myelolipomas are actually often misdiagnosed as liposarcomas [8].

Open surgery is a gold standard for the treatment of large myelolipomas. On the other hand, a transperitoneal or retroperitoneal approach with minimally invasive surgery is possible, as shown by Arezzo et al. [11]. Furthermore, Cochetti et al. showed that even large suprarenal myelolipomas can be treated by minimally invasive robotic surgery for the treatment of large adrenal myelolipoma [12]. Despite that, our decision for transabdominal open surgery was undertaken regarding the size of the tumor and easier approach to large vessels surrounding the tumor itself.

Currently, there are no consensus recommendations for postoperative surveillance of myelolipoma [9]. Classically, asymptomatic or small (<4 cm) myelolipomas are treated conservatively with routine cross-sectional surveillance imaging, while surgical resection is usually reserved for symptomatic or large (>7 cm) lesions [7]. On the other hand, low-grade liposarcomas, which myelolipoma could be mistaken for, are treated aggressively (extensive surgery is required) and have a worse prognosis.

Although myelolipomas are rare, and especially multiple extra-adrenal retroperitoneal types, we would like to remind the surgeons, radiologists, and pathologists to be aware of these tumors. The radiological evaluation supported by histological examination upon the fine needle biopsy is usually sufficient to establish the diagnosis, but in some cases of well-differentiated liposarcoma, telling the difference between myelolipoma and liposarcoma can be challenging. Therefore, considering that myelolipomas and liposarcomas have a diametrically opposite prognosis, which then drives the surgeon's decision on the treatment, we also emphasize the importance of intraoperative assessment of the tumor, both by the surgeon as well as by intraoperative pathological evaluation.

## Figures and Tables

**Table 1:** Extra-adrenal location of myelolipoma [2].

Location^∗^	
Head	Retroorbital
	Nasal cavity
	Mandible
Thorax	Endobronchial
	Pulmonary
	Pleural
	Mediastinal (posterior, anterior, middle)
	Chest wall
	Costal
Abdomen	Renal (hilum, sinus, extramedullary)
	Perirenal
	Periureteric
	Retroperitoneal
	Mesenterical (omental)
	Gastric
	Hepatic
	Splenic
	Perisplenic
Pelvis	Presacral
	Ovarian
	Lower pelvis
	Perivesical
Spinal	
Paravertebral	
Lymphonodal	

^∗^Myelolipomas can occur at location site or at multiple locations simultaneously.

## Data Availability

The data used to support the findings of this study are included within the article.
